# Effect of Xpert MTB/RIF on clinical outcomes in routine care settings: individual patient data meta-analysis

**DOI:** 10.1016/S2214-109X(18)30458-3

**Published:** 2019-02

**Authors:** Gian Luca Di Tanna, Ali Raza Khaki, Grant Theron, Kerrigan McCarthy, Helen Cox, Lucy Mupfumi, Anete Trajman, Lynn Sodai Zijenah, Peter Mason, Tsitsi Bandason, Betina Durovni, Wilbert Bara, Michael Hoelscher, Petra Clowes, Chacha Mangu, Duncan Chanda, Alexander Pym, Peter Mwaba, Frank Cobelens, Mark P Nicol, Keertan Dheda, Gavin Churchyard, Katherine Fielding, John Z Metcalfe

**Affiliations:** TB Centre, Riskcenter-IREA, Department of Econometrics, Statistics and Applied Economics, University of Barcelona, Barcelona, Spain; Division of Oncology, University of Washington, Seattle, WA, USA; DST-NRF Centre of Excellence for Biomedical Tuberculosis Research and SAMRC Centre for Tuberculosis Research, Division of Molecular Biology and Human Genetics, Faculty of Medicine and Health Sciences, Stellenbosch University, Tygerberg, South Africa; National Institute for Communicable Diseases, National Health Laboratory Service, Johannesburg, South Africa; Division of Medical Microbiology, and Institute of Infectious Disease and Molecular Medicine, University of Cape Town, Cape Town, South Africa; Botswana Harvard AIDS Institute, Gaborone, Botswana; Federal University of Rio de Janeiro, Rio de Janeiro, Brazil, McGill University, Montreal, QC, Canada; Department of Immunology, University of Zimbabwe College of Health Sciences, Harare, Zimbabwe; Biomedical Research and Training Institute, Harare, Zimbabwe; Biomedical Research and Training Institute, Harare, Zimbabwe; Municipal Health Secretariat, Rio de Janeiro, Brazil; Zimbabwe Ministry of Health and Child Welfare, Harare, Zimbabwe; Mbeya Medical Research Center, Mbeya, Tanzania, Division of Infectious Diseases and Tropical Medicine, Medical Center of the University of Munich, Munich, Germany; Mbeya Medical Research Center, Mbeya, Tanzania, Division of Infectious Diseases and Tropical Medicine, Medical Center of the University of Munich, Munich, Germany; Mbeya Medical Research Center, Mbeya, Tanzania; University Teaching Hospital and University of Zambia School of Medicine, Lusaka, Zambia; Africa Health Research Institute, Nelson R Mandela School of Medicine, University of KwaZulu-Natal, Durban, South Africa; University Teaching Hospital and University of Zambia School of Medicine, Lusaka, Zambia; Department of Global Health and Amsterdam Institute for Global Health and Development, Academic Medical Center, Amsterdam, Netherlands; National Institute for Communicable Diseases, National Health Laboratory Service, Johannesburg, South Africa, Division of Medical Microbiology, and Institute of Infectious Disease and Molecular Medicine, University of Cape Town, Cape Town, South Africa; London School of Hygiene and Tropical Medicine, London, UK, Lung Infection and Immunity Unit, Division of Pulmonology, University of Cape Town, Cape Town, South Africa; The Aurum Institute, Johannesburg, South Africa, School of Public Health, University of the Witwatersrand, Johannesburg, South Africa, Advancing Care and Treatment for TB/HIV, South African Medical Research Council, Johannesburg, South Africa; TB Centre; Division of Pulmonary and Critical Care Medicine, Zuckerberg San Francisco General Hospital, University of California, San Francisco, CA, USA

## Abstract

**Background:**

Xpert MTB/RIF, the most widely used automated nucleic acid amplification test for tuberculosis, is available in more than 130 countries. Although diagnostic accuracy is well documented, anticipated improvements in patient outcomes have not been clearly identified. We performed an individual patient data meta-analysis to examine improvements in patient outcomes associated with Xpert MTB/RIF.

**Methods:**

We searched PubMed, Embase, ClinicalTrials.gov, and the Pan African Clinical Trials Registry from inception to Feb 1, 2018, for randomised controlled trials (RCTs) comparing the use of Xpert MTB/RIF with sputum smear microscopy as tests for tuberculosis diagnosis in adults (aged 18 years or older). We excluded studies of patients with extrapulmonary tuberculosis, and studies in which mortality was not assessed. We used a two-stage approach for our primary analysis and a one-stage approach for the sensitivity analysis. To assess the primary outcome of cumulative 6-month all-cause mortality, we first performed logistic regression models (random effects for cluster randomised trials, with robust SEs for multicentre studies) for each trial, and then pooled the odds ratio (OR) estimates by a fixed-effects (inverse variance) or random-effects (Der Simonian Laird) meta-analysis. We adjusted for age and gender, and stratified by HIV status and previous tuberculosis-treatment history. The study protocol has been registered with PROSPERO, number CRD42014013394.

**Findings:**

Our search identified 387 studies, of which five RCTs were eligible for analysis. 8567 adult clinic attendees (4490 [63·5%] of 7074 participants for whom data were available were HIV-positive) were tested for tuberculosis with Xpert MTB/RIF (Xpert group) versus sputum smear microscopy (sputum smear group), across five low-income and middle-income countries (South Africa, Brazil, Zimbabwe, Zambia, and Tanzania). The primary outcome (reported in three studies) occurred in 182 (4·5%) of 4050 patients in the Xpert group and 217 (5·3%) of 4093 patients in the smear group (pooled adjusted OR 0·88, 95% CI 0·68–1·14 [p=0·34]; for HIV-positive individuals OR 0·83, 0·65–1·05 [p=0·12]). Kaplan-Meier estimates showed a lower rate of death (12·73 per 100 person-years in the Xpert group *vs* 16·38 per 100 person-years in the sputum smear group) for HIV-positive patients (hazard ratio 0·76, 95% CI 0·60–0·97; p=0·03). The risk of bias was assessed as reasonable and the statistical heterogeneity across studies was low (*I*^2^<20% for the primary outcome).

**Interpretation:**

Despite individual patient data analysis from five RCTs, we were unable to confidently rule in nor rule out an Xpert MTB/RIF-associated reduction in mortality among outpatients tested for tuberculosis. Reduction in mortality among HIV-positive patients in a secondary analysis suggests the possibility of population-level impact.

## Introduction

Tuberculosis is the leading global cause of death due to an infectious disease. Inadequate case detection is a major barrier to current tuberculosis control efforts, contributing to the 36% of the estimated 10 million cases in 2017 not notified to tuberculosis programmes.^1^ The poor sensitivity of sputum smear microscopy for the detection of acid-fast bacilli coupled with the laboratory infrastructure necessary for *Mycobacterium tuberculosis* culture provide strong impetus for the development of new, programme-relevant tuberculosis diagnostic tools.

The introduction of an automated nucleic acid amplification test (NAAT),^2^ the GeneXpert MTB/RIF assay (also known as Xpert MTB/RIF; Cepheid, Sunnyvale, CA, USA) in December, 2010, was a groundbreaking advance in tuberculosis diagnostics. Relative to the conventional standard (ie, sputum smear microscopy), Xpert MTB/RIF offers improved analytic sensitivity and high specificity for individuals not previously treated for tuberculosis, and allows for drug susceptibility testing for rifampicin resistance with an on-demand (ie, random access, without need for batching) quality-controlled system requiring only minimal laboratory technician training. However, international scale-up and optimal programmatic use of Xpert MTB/RIF has been delayed by high product costs, inadequate service and maintenance plans, inability to use the platform within most microscopy centres, and lack of attention to dedicated implementation strategies.^3–5^ Furthermore, despite the success of demonstration studies^6,7^ and early cost-effectiveness models adopting treatment benefits derived from observational studies,^8,9^ evidence for improved morbidity and mortality with Xpert MTB/RIF has thus far not been supported by high-quality studies.

As in randomised controlled trials (RCTs) of tuberculosis therapeutics,^10^ surrogate markers for patient-important outcomes for diagnostic-test evaluation—such as test accuracy, turnaround time, time to diagnosis, and time to treatment—might not translate onward to clinical outcomes and could be subject to substantial hetero-geneity.^11^ Although therapeutic outcomes are similarly of primary interest in diagnostic RCTs,^12,13^ they have received less methodological attention than tuberculosis therapeutic trials.^14^ Five diagnostic RCTs^15–19^ across 41 sites in South Africa, Brazil, Zimbabwe, Zambia, and Tanzania, assessing the effect of Xpert among people tested for tuberculosis in routine-care settings have been done. Along with consistent increases in the proportion of bacteriologically confirmed cases, the possibility of decreased early mortality at 2 months^15^ and 3 months^16^ has been raised, although estimates have been imprecise. Overall, high rates of empirical tuberculosis treatment, a relatively low-risk target population (eg, outpatients), and type 2 error (ie, insufficient power) have been most commonly used to explain the absence of effect on morbidity or mortality.^20^

We did a systematic review and an individual patient data (IPD) meta-analysis to examine whether use of Xpert MTB/RIF as a replacement for conventional sputum smear microscopy improves patient-centred outcomes such as mortality and time to tuberculosis diagnosis and treatment. Furthermore, we assessed the possibility of differential effects across prespecified patient subgroups, including HIV-positive individuals and those with a previous history of tuberculosis treatment.

## Methods

### Search strategy and selection criteria

For this systematic review and meta-analysis we searched PubMed, Embase, ClinicalTrials.gov, and the Pan African Clinical Trials Registry from inception to Feb 1, 2018, for RCTs in English; we also contacted experts in the field to identify ongoing and completed trials. A search strategy was developed in consultation with an information specialist (librarian) who has extensive experience in systematic reviews. Search terms included a combination of free text and controlled vocabulary (ie, Medical Subject Headings terms): ((tuberculosis AND (mtb/rif OR xpert OR genexpert OR cepheid OR NAAT OR NAA test* OR nucleic acid amplification test*)) OR (tuberculosis[mh] AND “nucleic acid amplification techniques”[mh])) AND (“randomized controlled trial”[pt] OR random* OR “controlled trial”). Additional terms included: “tuberculosis”, “MTB/ RIF”, “Xpert”, “GeneXpert”, “Cepheid”, “nucleic acid amplification test”, “NAAT”, and “randomized controlled trial”. We excluded studies that were not RCTs; did not use Xpert or sputum smear microscopy; included children, patients with extrapulmonary tuberculosis, or patients not tested for tuberculosis; did not compare Xpert and sputum smear microscopy; did not assess mortality. ARK and JZM did the search and ARK and GLDT extracted data. We used IPD for participants aged 18 years and older from randomised trials that, to our knowledge, represented the totality of evidence from RCTs evaluating the effect of Xpert MTB/ RIF versus sputum smear microscopy on patient outcomes among outpatients tested for tuberculosis.

### Data analysis

Data were extracted for age, gender, HIV, history of tuberculosis, clinical symptoms, and body-mass index (BMI) or weight, where BMI was not available. Data were quality checked for each individual trial (all potential duplicates and missing data were assessed with the trial contributors) and then assembled to constitute a master data file.

Our primary outcome was cumulative risk (expressed as odds ratio [OR]) of all-cause mortality at 6 months following random assignment to groups of clinic attendees tested for tuberculosis with Xpert MTB/RIF (Xpert group) versus sputum-smear microscopy (sputum-smear group). Secondary outcomes were time to death, time to tuberculosis diagnosis (ie, time from specimen collection to result available in the laboratory, if bacteriologically confirmed), time to tuberculosis treatment (bacteriologically or clinically confirmed), and 3-month cumulative mortality risk. We calculated the hazard ratio (HR) for time-to-event analyses and the OR for the 3-month mortality risk. Separately, among clinic attendees investigated for tuberculosis who started receiving tuberculosis treatment, we analysed time to death while on treatment. We prespecified stratified analyses for each outcome by HIV status and by history of previous tuberculosis treatment. Outcomes for which each study contributed data are summarised in the appendix. Risk of bias was assessed using the Cochrane Risk of Bias tool by two authors (ARK and JZM).

We did IPD meta-analysis using both one-stage and two-stage approaches.^21^ We report our primary analysis using the two-stage approach, and a sensitivity analysis using the one-stage approach. In the two-stage approach, for the binary outcomes (all-cause mortality at 3 months and 6 months), we fitted a logistic regression model for each study. Robust SEs were calculated for any multicentre study and a random-effects (at cluster level) logistic model was used for cluster-randomised trials. Pooled estimates were then obtained by fixed-effects (inverse variance) or random-effects (Der Simonian Laird) meta-analysis; we reported the results of random-effects meta-analysis when statistical heterogeneity, assessed through a formal test of homogeneity and *I*^2^, was large (approximately >60%). For time-to-event outcomes, we used proportional-hazards Cox models, with robust SEs for multicentre studies and a shared frailty (random effect for cluster) for cluster-randomised trials. For stepped-wedge trials, we used a random-effects (laboratory-level) Cox model with a fixed effect for a covariate set including the timepoints at which Xpert MTB/RIF was introduced. All analyses were adjusted for age and gender; estimates of the pooled-intervention effect were further adjusted for weight (as a proxy for BMI) and number of tuberculosis symptoms (as a categorical variable), when possible. In the one-stage approach, we combined all IPD in a single meta-analysis,^22^ with a hierarchical logistic regression allowing for study and cluster (if relevant) random effects for the binary outcomes. Cox models were fitted with shared frailties at study level and cluster level (if relevant) for time-to-event outcomes. For time-to-tuberculosis diagnosis, a follow-up time of 0·5 days was assumed for patients diagnosed on the same day as sputum collection. Only specimens taken at enrolment were used to define the time-to-tuberculosis diagnosis outcome. Data management and statistical analyses were done with Stata 14.

We prespecified our analytical plan in accordance with international recommendations^23^ and registered the study protocol with PROSPERO (CRD42014013394).

### Role of the funding source

The funder of the study had no role in study design, data collection, data analysis, data interpretation, or writing of the report. The corresponding author had full access to all the data in the study and had final responsibility for the decision to submit for publication.

## Results

**Figure 1:**
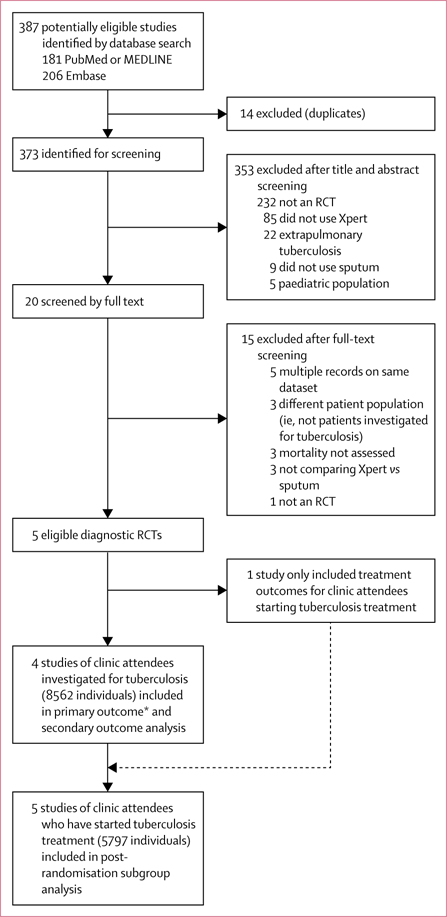
Study selection RCT=randomised controlled trial. *The primary outcome was limited to three studies^15,17,18^ (n=8142), as Mupfumi and colleagues^16^ limited follow-up to 3 months.

Our database search identified 387 potentially eligible studies. After the exclusion of 14 duplicates, of 353 studies after title and abstract screening, and of 15 studies after whole-text screening, we found five eligible RCTs comparing the use of Xpert MTB/RIF versus sputum smear microscopy for the diagnosis of tuberculosis (figure 1).^15–19^

All trials included participants aged 18 years or older. The XTEND study^18^ was a cluster-randomised trial randomly allocating 20 laboratories in medium-burden districts in four South African provinces (n=4656 patients) to assess 6-month mortality among clinic attendees being tested for tuberculosis. Using weeks randomly allocated to Xpert or sputum-smear groups, Cox and colleagues^17^ analysed 1985 patients at a single primary health-care clinic in Khayelitsha, Cape Town, South Africa, and assessed the proportion of patients with bacteriologically confirmed tuberculosis who had not started receiving appropriate tuberculosis treatment within 2 months after enrolment. Mupfumi and colleagues^16^ randomly assigned 424 HIV-positive patients initiating antiretroviral therapy at a single centre in Harare, Zimbabwe, to be tested for tuberculosis with either Xpert MTB/RIF or sputum smear microscopy, and examined a composite endpoint of 3-month mortality and antiretroviral therapy-associated tuberculosis (ie, unmasking of subclinical tuberculosis disease). The TB-NEAT study^15^ randomly assigned to either Xpert or sputum-smear groups 1502 patients attending primary care clinics in South Africa, Tanzania, Zambia, and Zimbabwe who had at least one tuberculosis symptom (two if HIV-negative) and were able to expectorate two sputum specimens; the primary outcome was tuberculosis-related morbidity at 2 months and 6 months. Durovni and colleagues^19^ did a stepped-wedge cluster-randomised trial (CRT) in Rio de Janeiro and Manaus, Brazil, randomising the sequence of Xpert MTB/RIF introduction in 14 laboratories (24 227 patients) and assessing the notification proportion of laboratory-confirmed pulmonary tuberculosis. A second publication^24^ from the same research group reported mortality outcomes among patients who were initiated on tuberculosis treatment in the primary trial; these data contributed to the current analysis.

Our patient-level pooled analysis included data from 8567 adult outpatients tested for tuberculosis. Participant characteristics, stratified by trial and according to initial randomisation (ie, intention to treat), are shown in table 1. Overall HIV-positivity in these trials was 63·5% (4490 of the 7074 participants tested for HIV; ranging from 59%^17^ to 100%^16^); overall, 54·5% (4665 of 8567) of participants were women and the median age was 37 years (IQR 29–47 years). Except for the inability to blind the intervention (for all trials), and lack of allocation concealment (for two trials),^17,19^ risk of bias was assessed to be reasonable and the statistical heterogeneity across studies was generally low (*I*^*2*^ <20% for the mortality outcomes; appendix). For the primary outcome of 6-month mortality risk among outpatients tested for tuberculosis, 399 (4·9%) primary endpoints among 8142 individuals (three studies; figure 1)^15,17,18^ contributed to the analysis. Overall all-cause 6-month mortality occurred in 182 (4·5%) of 4050 patients in the Xpert group and 217 (5·3%) of 4093 patients in the sputum-smear group (pooled OR 0·88, 95% CI 0·68–1·14; p=0·34); stratified analysis showed a pooled OR of 0·83 (0·65–1·05; p=0·12) for HIV-positive individuals and 0·83 (0·46–1·5; p=0·55) for HIV-negative individuals. Analysis of 3-month mortality (four studies)^15–18^ and stratification by history of previous tuberculosis gave results similar to the overall estimate (table 2).

**Figure 2:**
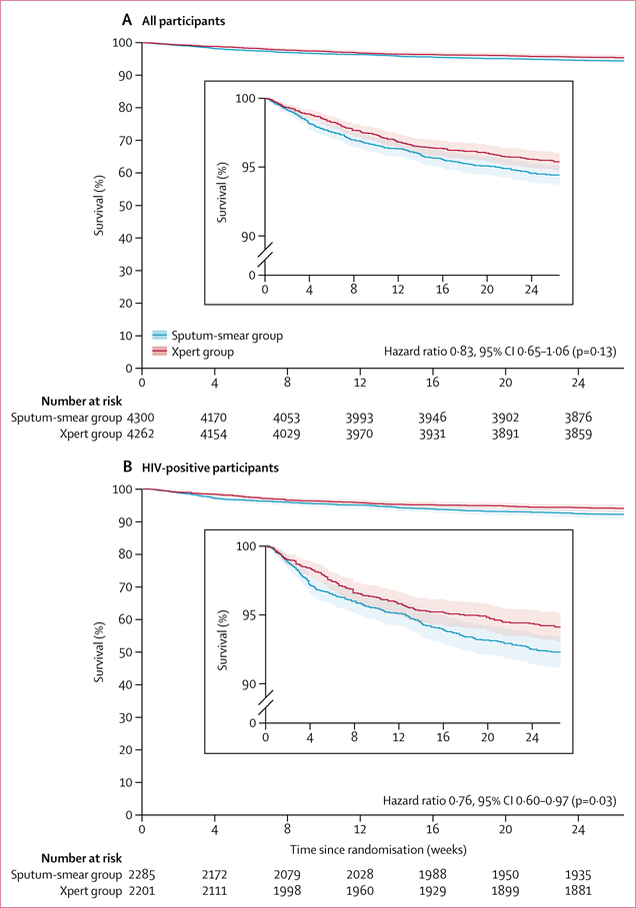
Kaplan-Meier pooled survival estimates Shaded areas represent 95% CIs.

Time to death among clinic attendees investigated for tuberculosis included 425 (5·0%) events among 8561 individuals (four studies)^15–18^ over 3983 person-years of follow-up. With time-to-event analysis, there were 9·69 deaths per 100 person-years in the Xpert group and 11·63 deaths per 100 person-years in the smear microscopy group (HR 0·83, 95% CI 0·65–1·06; p=0·13; figure 2). These results were similar in sensitivity analyses adjusting for age, gender, weight, and tuberculosis symptoms (6568 individuals from three studies;^15,16,18^ appendix). Among HIV-positive individuals, all-cause death in the Xpert group (12·73 per 100 person-years) was lower than in the sputum-smear group (16·38 per 100 person-years; HR 0·76, 0·60–0·97; p=0·03) as per the data from four studies (figure 2).^15–18^ The significance of this finding was maintained after further adjustment for age, gender, weight, and tuberculosis symptoms (appendix).

Across all trials, 6468 (19·7%; ranging from 12%^18^ to 43%^15^) of 32 794 outpatients investigated for tuberculosis ultimately received tuberculosis treatment. Time to tuberculosis diagnosis did not differ for 1924 individuals from two studies, with a median of 0·5 days (IQR 0·5–10) for each group (pooled HR 1·05, 95% CI 0·93–1·19; p=0·43).^15,16^ The median time to tuberculosis treatment for 8208 individuals from four studies^15–18^ was 4 days (1–10) for the Xpert group versus 5 days (1–15) for the smear group (1·0, 0·75–1·32; p=0·99), although it was shorter for patients in the Xpert group reporting a history of previous tuberculosis treatment than for patients in the sputum-smear group (0·73, 95% CI 0·53–0·99; p=0·04). In a prespecified subgroup time-to-event analysis of 5797 adults who started receiving tuberculosis treatment (all trials),^15–19^ we found a possible 33% relative decrease in the rate of deaths in the Xpert group versus the sputum-smear group (0·67, 0·50–0·90; p=0·007). 6-month all-cause mortality was higher among individuals tested for tuberculosis but who ultimately did not start receiving treatment than among those initiating tuberculosis treatment (4·3% [133 of 3082] in the Xpert group *vs* 6·7% [182/2715] in the sputum-smear group). In this group of individuals, the summary HR for death in the Xpert group versus sputum-smear group was 0·89 (0·70–1·14; p=0·34) among all outpatients, and 0·84 (0·67–1·05; p=0·12) among HIV-positive individuals.

## Discussion

In an IPD meta-analysis of 8567 outpatients tested for tuberculosis in five low-income and middle-income countries, we were unable to rule in nor rule out a reduction in 6-month all-cause mortality associated with use of Xpert MTB/RIF as an initial diagnostic test, relative to sputum smear microscopy. Our results are consistent with a plausible reduction in mortality of up to 32% and up to a 14% increase in mortality, with the best estimate being a 12% reduction. Xpert MTB/RIF use was not associated with reduced time to tuberculosis diagnosis or to commencement of tuberculosis treatment, nor with an increased proportion of individuals treated for tuberculosis. We did, however, find modest evidence for decreased HIV-specific mortality.

Unlike HIV and malaria—other major global causes of death and morbidity from infectious disease—there is no simple and affordable point-of-care test for tuberculosis. In a widely cited decision tree model, Keeler and colleagues^25^ estimated over a decade ago that a new rapid tuberculosis diagnostic test with 89% sensitivity and 99% specificity, accessible in clinics and hospitals, would prevent some 200 000 tuberculosis deaths annually, or about 10% of tuberculosis mortality. Investments in Xpert MTB/RIF have accounted for a substantial proportion of global tuberculosis diagnostics spending since WHO endorsement in December, 2010, with 6·9 million Xpert MTB/RIF cartridges procured in the public sector of 130 of the 145 countries eligible for concessional pricing in 2016 alone.^26^

A clear effect on patient mortality with programmatic use of Xpert MTB/RIF might be difficult to detect for several reasons. First, losses within the passive case-finding cascade during the prediagnostic, or even pretreatment,^27^ periods might not be greatly improved by Xpert MTB/RIF. Symptomatic or asymptomatic individuals with active tuberculosis who never access care or who are not appropriately triaged account for a large proportion of the estimated 3·6 million globally undetected cases each year, presumably due to operational health-system weaknesses. Although not included in our analytic population, this group remains an important focus for tuberculosis programmes and for the implementation of new diagnostic strategies in low-income and middle-income countries. Basic, translational, and operational research towards a simple, affordable, truly point-of-care test with high analytical sensitivity for tuberculosis remains greatly needed. Second, the effect of empirical treatment in biasing toward the null in diagnostics studies has been extensively discussed.^20,28,29^ The decision to initiate treatment in the absence of bacteriological confirmation is complex, as it varies by setting,^30^ patient (eg, highest risk patients might be most likely to benefit from early, accurate diagnosis, but also more likely to be empirically treated), time since test introduction (eg, enthusiasm after Xpert MTB/RIF training among South African National Department of Health staff might have led to baseline imbalances in the XTEND trial^18^ that favoured Xpert MTB/RIF, and empirical treatment has declined substantially over time in at least one setting in South Africa, possibly an evolving adaptation to Xpert MTB/RIF availability),^31^ and—probably—trial effects.^32^ Overall, in the largest analysed studies^15,18,19^ and in a 2017 CRT^33^ of centralised versus on-site testing, Xpert MTB/RIF did not significantly increase the proportion of patients treated for tuberculosis. Finally, insufficient power (ie, leading to type 2 error—the risk that a treatment benefit will not be shown, even if it exists) is perhaps the most common explanation for failure to reach a prespecified primary outcome.^34^ Although post-hoc power curves might defy interpretation,^36^ to observe a 12% relative mortality reduction with use of Xpert MTB/RIF (eg, a reduction from 8% to 7% absolute mortality, consistent with our reported point estimate) with 90% power would require 16 064 people per group, not accounting for clustering.

Nevertheless, a prespecified secondary subgroup analysis did suggest a mortality benefit among HIV-positive individuals. HIV-associated tuberculosis was one of two priority groups (along with individuals investigated for drug-resistant tuberculosis) in the initial 2011 WHO Xpert MTB/RIF policy statement,^2^ and high relative mortality, particularly among those who are antiretroviral treatment naive, increases the statistical power to detect an effect in this group. Although a secondary outcome, time-to-death analysis included a larger number of individuals and a more complete covariate adjustment than 6-month cumulative mortality-risk analysis (our primary outcome), leading to increased power. Biological plausibility, consistency with diagnostic accuracy,^37^ with modelling data,^38^ and with the results of a CRT^39^ of tuberculosis screening among adults newly diagnosed with HIV in Malawi, reinforce this secondary analysis; therefore, we consider this result as overall modest evidence that should be replicated.

In a prespecified subgroup analysis, we found that individuals tested with Xpert MTB/RIF and who—regardless of test result—subsequently started tuberculosis treatment had lower mortality than those tested with sputum smear microscopy. Interpretation of this finding is complex, and is a function of our conception of how these groups are related and of perceived direction of bias. Enhanced analytical sensitivity of Xpert MTB/RIF could allow the diagnosis of more individuals at an earlier stage in their disease process, or the loss of fewer individuals who have a milder disease within the diagnostic cascade, accounting for the noted effect (ie, lower mortality), than is possible with sputum smear microscopy. Alternatively, unmeasured confounding from a factor unrelated to diagnostic-test performance but differentially distributed among those initiating treatment after Xpert MTB/RIF versus sputum smear microscopy might account for this apparent association. Although arguably the principal mediator of the effect of tuberculosis diagnostic test on death and other adverse outcomes, tuberculosis treatment is a post-randomisation process. Therefore, the assumption of sequential ignorability (ie, that tuberculosis treatment is effectively randomly assigned given baseline covariates and the randomly allocated diagnostic group) cannot be assumed. For this reason, we present these results primarily as hypothesis generating.

Our IPD meta-analysis had several limitations. First, complete patient-level data were not available for CD4-positive T-lymphocyte counts, tuberculosis symptoms, and BMI for all studies, precluding full adjustment for these key covariates, with tuberculosis symptoms and BMI being important covariates in the XTEND study.^18^ Second, we report analysis of our data primarily using a two-stage meta-analytic approach, because this was required for inclusion of time-phase as a covariate for the stepped-wedge trial included in this meta-analysis.^19^ Acknowledging that the one-stage approach better accounts for potential risk of ecological bias than the two-stage approach,^40^ no convincing evidence exists for the supremacy of one-stage versus two-stage approaches. Third, our study results might be less applicable to Xpert Ultra, which has a lower limit of detection than Xpert MTB/RIF.^41^ Fourth, although the XTEND trial^18^ was analysed according to a cluster-level summary approach in its primary publication—as recommended for a small number of clusters^42^—we analysed XTEND herein using random effects; we cannot exclude the possibility that this analysis might have led to non-robust intervention-effect estimates for the XTEND study. Finally, our analytical population excluded important patient groups who are likely to draw particular benefit from Xpert MTB/RIF, including children and those with rifampicin-resistant tuberculosis.

In conclusion, in this large multicountry IPD meta-analysis, we could neither rule in nor rule out a reduction in mortality over a 6-month period among outpatients tested for tuberculosis with Xpert MTB/RIF rather than with sputum smear microscopy. Our finding of a potential mortality benefit among HIV-positive individuals in our secondary outcome of time to death provides justification for trials of novel tuberculosis diagnostics in this or other high-risk groups.

## Supplementary Material

1

## Figures and Tables

**Table 1:** Baseline participant characteristics

	Theron et al (2014)^15^	Mupfumi et al (2014)^16^	Cox et al (2014)^17^	Churchyard et al (2015)^18^	Durovni et al (2014)^19^ and Trajman et al (2015)^22^
**Trial characteristics**					
Type of trial	RCT	RCT	CRT	CRT	SW
Comparative SOC*	FM†	FM	FM	FM	ZN
Setting	South Africa, Zimbabwe, Zambia, Tanzania	Harare (Zimbabwe)	Khayelitsha (South Africa)	South Africa	Brazil
**Adults investigated for tuberculosis**					
Analytic population, n	1502	424	1985	4656	NA
Xpert MTB/RIF group, n (%)	744 (50%)	214 (51%)	982 (50%)	2324 (50%)	NA
Age (SD), years	39 (12)	38 (10)	41 (12)	38 (13)	NA
Female, n (%)	643 (43%)	232 (55%)	900 (45%)	2890 (62%)	NA
BMI (SD), kg/m^2^	21·9 (5·2)	NR	NR	25·5 (6·9)	NA
Weight (SD), kg	59·7 (12·9)	59·7 (11·7)	NR	65·1 (15·0)	NA
Any tuberculosis symptoms, n (%)	1497 (99·9%)	388 (92%)	NR	4382 (94%)	NA
Mean number of tuberculosis symptoms (SD)	3·2 (0·9)	2·3 (1·3)	NR	2·9 (1·3)	NA
Prevalence of previous history of tuberculosis, n (%)	365 (24%)	52 (12%)	NR	718 (15%)	NA
HIV-positive individuals, n/N (%)‡	895/1483 (60%)	424/424 (100%)	965/1625 (59%)	2206/3542 (62%)	NA
Patients with CD4 ≥100 cells per µL, n/N (%)‡	582/861 (68%)	272/420 (65%)	NR	998/1130 (88%)	NA
Overall trial mortality, n/N (%)	121/1502 (8%)	28/354 (8%)§	71/1985 (4%)	207/4608 (5%)¶	NA
**Adults investigated for tuberculosis and initiated on treatment**					
Population of adults on tuberculosis treatment n/N (%)	647/1502 (43%)	100/424 (24%)	540/1985 (27%)	541/4656 (12%)	4640/24 227 (19·2%)
Analytic population, n||	647	84	506	520	4088
Xpert MTB/RIF group, n (%)	315 (49%)	41 (49%)	277 (55%)	240 (46%)	2232 (55%)
Age (SD), years	38·9 (11·8)	36·9 (9·7)	37·7 (10·1)	37·9 (11·8)	**
Female, n (%)	289 (45%)	32 (38%)	306 (61%)	225 (43%)	1444 (35%)
BMI (SD), kg/m^2^	21·8 (5·2)	NR	NR	22·4 (5·5)	NR
Weight (SD), kg	59·4 (13·0)	56·1 (9·3)	NR	58·6 (11·3)	NR
Any tuberculosis symptoms, n (%)	645 (100%)	84 (100%)	NR	514 (99%)	NR
Mean number of tuberculosis symptoms (SD)	3·1 (0·9)	3·1 (1·0)	NR	3·4 (1·0)	NR
Individuals with previous history of tuberculosis, n (%)	155 (24%)	11 (13%)	NR	89 (17%)	NR
HIV-positive individuals, n/N (%)‡	402/639 (63%)	84/84 (100%)	298/506 (59%)	267/373 (72%)	399/2040 (20%)
Patients with CD4 ≥100 cells per µL, n/N (%)‡	262/376 (70%)	43/84 (51%)	NR	90/116 (78%)	NR

RCT=individually randomised controlled trial. CRT=cluster randomised trial. SW=stepped wedge. SOC=standard of care. FM=fluorescent microscopy with auramine staining. ZN=direct light microscopy with Ziehl-Neelsen staining. NA=not applicable. BMI=body-mass index. NR=not reported.

* All trials assessed an intervention group of a single sputum specimen analysed with Xpert MTB/RIF (Generation 3; Cepheid, Sunnyvale, CA, USA) in close central laboratories, with the exception of TB-NEAT,^15^ in which Xpert MTB/RIF was done on site; all trials assessed a SOC group of a single sputum specimen analysed by sputum smear microscopy as noted, except XTEND,^18^ which examined two sputum specimens.

† One of five sites (ie, Ifisi Day Clinic in Mbeya, Tanzania) used ZN microscopy.

‡ HIV status and CD4 cell count were unknown for a certain proportion of participants in some trials.

§ Denominator used to calculate mortality was the total number of patients for whom the outcome could be assessed at 3 months from randomisation.

¶ Vital status at 6 months from enrolment was unknown for 48 participants.

|| Analysis populations might differ from total population initiating treatment due to missing data or loss to follow-up.

** Age analysed as a categorical variable.

**Table 2:** Effect of Xpert MTB/RIF on patient outcomes relative to smear microscopy

	Number of adults	OR or HR (95% CI)	p value	*I*^2^	p (test of homogeneity)
**Adults investigated for tuberculosis**					
6-month mortality risk^15,17,18*^					
Overall	8142	0·88 (0·68–1·14)	0·34	0·0%	0·94
HIV negative	2583	0·83 (0·46–1·5)	0·55	0·0%	0·83
HIV positive	4066	0·83 (0·65–1·05)	0·12	14·8%	0·31
No history of tuberculosis	6519	0·95 (0·76–1·19)	0·67	0·0%	0·58
History of tuberculosis	1623	0·91 (0·51–1·61)	0·74	0·0%	0·39
Time to death					
Overall^15–18^	8561	0·83 (0·65–1·06)	0·13	0·0%	0·75
HIV negative^15,17,18^	2583	0·81 (0·46–1·41)	0·45	0·0%	0·80
HIV positive^15–18^	4486†	0·76 (0·60–0·97)	0·03	0·0%	0·47
No history of tuberculosis^15–18^	6887	0·87 (0·68–1·1)	0·24	0·0%	0·79
History of tuberculosis^15–18^	1674	0·83 (0·49–1·42)	0·50	0·0%	0·46
3-month mortality risk					
Overall^15–18^	8566	0·91 (0·68–1·20)	0·50	0·0%	0·64
HIV-negative^15,17,18^	2583	0·74 (0·38–1·45)	0·38	0·0%	0·94
HIV-positive^15–18^	4490	0·86 (0·66–1·13)	0·29	0·0%	0·46
No history of tuberculosis^15–18^	6891	1·04 (0·80–1·35)	0·77	0·0%	0·57
History of tuberculosis^15–18^	1675	0·79 (0·40–1·58)	0·51	0·0%	0·49
Time to tuberculosis diagnosis^15,16‡^					
Overall	1924	1·05 (0·93–1·19)	0·43	47·5%	0·17
HIV positive	1164	0·99 (0·86–1·16)	>0·99	25·2%	0·25
Time to tuberculosis treatment					
Overall^15–18‡^	8208	1·00 (0·75–1·32)	0·99	85·4%	<0·0001
HIV negative^15,17,18‡^	2482	0·88 (0·60–1·30)	0·52	60·2%	0·08
HIV positive^15–18‡^	4251	1·04 (0·76–1·42)	0·80	86·0%	<0·0001
No history of tuberculosis^15,16,18‡^	5213	0·9 (0·70–1·17)	0·44	70·0%	0·04
History of tuberculosis^15,16,18^	1074	0·73 (0·53–0·99)	0·04	0·0%	0·63
**Adults investigated for tuberculosis and initiated on treatment**					
Time to death					
Overall^15–19^	5797	0·67 (0·50–0·90)	0·007	0·0%	0·63
HIV negative^15,17–19^	2670	0·55 (0·3–1·02)	0·06	0·0%	0·79
HIV positive^15–19^	1445	0·85 (0·55–1·31)	0·46	18·0%	0·30

All pooled estimates are adjusted for age and gender and are reported with a two-stage analytic approach with fixed effects, unless heterogeneity (*I*^2^) was high (ie, *I*^2^>60%), in which case random effects are primarily reported. Sensitivity analyses with random effects regardless of heterogeneity (*I*^2^) estimate, additional covariate adjustment, and a one-stage analytical approach are reported in the appendix. ORs are reported for 6-month and 3-month mortality risks. HR reported for all time-to-event analyses. OR=odds ratio. HR=hazard ratio.

* 6-month mortality risk was the primary outcome.

† Five individuals without date of death were excluded.

‡ Random effects used to combine study estimates, as *I*^2^ was large.

## Research in context

### Evidence before this study

Relative to acid-fast bacilli sputum smear microscopy, the first *Mycobacterium tuberculosis*-specific automated nucleic acid amplification test—GeneXpert MTB/RIF (Xpert MTB/RIF)—has shown improvement in several key surrogate patient outcomes, such as analytical sensitivity, test turnaround time, time to treatment initiation, and increase in proportion of bacteriologically confirmed cases. However, randomised controlled trials among clinic attendees tested for tuberculosis in routine-care settings, the endpoints of which have included morbidity and mortality, have produced inconclusive results. We hypothesised that pooling these trials in an individual patient data meta-analysis would provide increased power in determining whether Xpert MTB/RIF does confer such downstream benefits.

### Added value of this study

By pooling patient-level data from five diagnostic randomised controlled trials (n=8567 patients) in 41 sites in South Africa, Brazil, Zimbabwe, Zambia, and Tanzania, we found that use of Xpert MTB/RIF was not associated with decreased time to either tuberculosis diagnosis or commencement of tuberculosis treatment, and did not decisively reduce overall 6-month all-cause mortality among outpatients investigated for tuberculosis, relative to smear microscopy; our results are consistent with a possible mortality reduction of up to 32% and a mortality increase of up to 14%, with the best estimate being a 12% reduction. There was modest evidence for a decrease in HIV-specific mortality.

### Implications of all the available evidence

We were unable to conclusively show Xpert MTB/RIF-associated reduction in mortality among clinic attendees tested for tuberculosis relative to sputum smear microscopy. There remains uncertainty on this question, in particular around a potential mortality benefit among HIV-positive individuals or other high-risk groups. The impact of novel diagnostics on overall mortality is likely to remain attenuated in the context of sustained health system weaknesses.

## References

[1] WHO. Global tuberculosis report 2018 2018 http://www.who.int/tb/publications/global_report/en/ (accessed on Dec 1, 2018).

[2] WHO. Automated real-time nucleic acid amplification technology for rapid and simultaneous detection of tuberculosis and rifampicin resistance: Xpert MTB/RIF system. Policy statement Geneva: World Health Organization, 2011. 26158191

[3] Albert H, Nathavitharana RR, Isaacs C, Pai M, Denkinger CM, Boehme CC. Development, roll-out and impact of Xpert MTB/RIF for tuberculosis: what lessons have we learnt and how can we do better? Eur Respir J 2016; 48: 516–25. 27418550 10.1183/13993003.00543-2016 PMC4967565

[4] Creswell J, Codlin AJ, Andre E, Results from early programmatic implementation of Xpert MTB/RIF testing in nine countries. BMC Infect Dis 2014; 14: 2. 24383553 10.1186/1471-2334-14-2 PMC3898850

[5] Hanrahan CF, Haguma P, Ochom E, Implementation of Xpert MTB/RIF in Uganda: missed opportunities to improve diagnosis of tuberculosis. Open Forum Infect Dis 2016; 3: ofw068. 27186589 10.1093/ofid/ofw068 PMC4866550

[6] Boehme CC, Nabeta P, Hillemann D, Rapid molecular detection of tuberculosis and rifampin resistance. N Engl J Med 2010; 363: 1005–15. 20825313 10.1056/NEJMoa0907847 PMC2947799

[7] Boehme CC, Nicol MP, Nabeta P, Feasibility, diagnostic accuracy, and effectiveness of decentralised use of the Xpert MTB/RIF test for diagnosis of tuberculosis and multidrug resistance: a multicentre implementation study. Lancet 2011; 377: 1495–505. 21507477 10.1016/S0140-6736(11)60438-8 PMC3085933

[8] Pantoja A, Fitzpatrick C, Vassall A, Weyer K, Floyd K. Xpert MTB/RIF for diagnosis of tuberculosis and drug-resistant tuberculosis: a cost and affordability analysis. Eur Respir J 2013; 42: 708–20. 23258774 10.1183/09031936.00147912

[9] Vassall A, van Kampen S, Sohn H, Rapid diagnosis of tuberculosis with the Xpert MTB/RIF assay in high burden countries: a cost-effectiveness analysis. PLoS Med 2011; 8: e1001120. 22087078 10.1371/journal.pmed.1001120 PMC3210757

[10] Warner DF, Mizrahi V. Shortening treatment for tuberculosis— back to basics. N Engl J Med 2014; 371: 1642–43. 25337754 10.1056/NEJMe1410977

[11] Naaktgeboren CA, Ochodo EA, Van Enst WA, Assessing variability in results in systematic reviews of diagnostic studies. BMC Med Res Methodol 2016; 16: 6. 26772804 10.1186/s12874-016-0108-4 PMC4714528

[12] Bossuyt PM, Reitsma JB, Linnet K, Moons KG. Beyond diagnostic accuracy: the clinical utility of diagnostic tests. Clin Chem 2012; 58: 1636–43. 22730450 10.1373/clinchem.2012.182576

[13] Squire SB, Ramsay AR, van den Hof S, Making innovations accessible to the poor through implementation research. Int J Tuberc Lung Dis 2011; 15: 862–70. 21682960 10.5588/ijtld.11.0161

[14] Lijmer JG, Bossuyt PM. Various randomized designs can be used to evaluate medical tests. J Clin Epidemiol 2009; 62: 364–73. 18945590 10.1016/j.jclinepi.2008.06.017

[15] Theron G, Zijenah L, Chanda D, Feasibility, accuracy, and clinical effect of point-of-care Xpert MTB/RIF testing for tuberculosis in primary-care settings in Africa: a multicentre, randomised, controlled trial. Lancet 2014; 383: 424–35. 24176144 10.1016/S0140-6736(13)62073-5

[16] Mupfumi L, Makamure B, Chirehwa M, Impact of Xpert MTB/ RIF on antiretroviral therapy-associated tuberculosis and mortality: a pragmatic randomized controlled trial. Open Forum Infect Dis 2014; 1: ofu038. 25734106 10.1093/ofid/ofu038 PMC4324195

[17] Cox HS, Mbhele S, Mohess N, Impact of Xpert MTB/RIF for tuberculosis diagnosis in a primary care clinic with high tuberculosis and HIV prevalence in South Africa: a pragmatic randomised trial. PLoS Med 2014; 11: e1001760. 25423041 10.1371/journal.pmed.1001760 PMC4244039

[18] Churchyard GJ, Stevens WS, Mametja LD, Xpert MTB/RIF versus sputum microscopy as the initial diagnostic test for tuberculosis: a cluster-randomised trial embedded in South African roll-out of Xpert MTB/RIF. Lancet Glob Health 2015; 3: e450–57. 26187490 10.1016/S2214-109X(15)00100-X

[19] Durovni B, Saraceni V, van den Hof S, Impact of replacing smear microscopy with Xpert MTB/RIF for diagnosing tuberculosis in Brazil: a stepped-wedge cluster-randomized trial. PLoS Med 2014; 11: e1001766. 25490549 10.1371/journal.pmed.1001766 PMC4260794

[20] Auld AF, Fielding KL, Gupta-Wright A, Lawn SD. Xpert MTB/RIF— why the lack of morbidity and mortality impact in intervention trials? Trans R Soc Trop Med Hyg 2016; 110: 432–44. 27638038 10.1093/trstmh/trw056

[21] Burke DL, Ensor J, Riley RD. Meta-analysis using individual participant data: one-stage and two-stage approaches, and why they may differ. Stat Med 2017; 36: 855–75. 27747915 10.1002/sim.7141 PMC5297998

[22] Riley RD, Lambert PC, Abo-Zaid G. Meta-analysis of individual participant data: rationale, conduct, and reporting. BMJ 2010; 340: c221. 20139215 10.1136/bmj.c221

[23] Tierney JF, Vale C, Riley R, Individual participant data (IPD) meta-analyses of randomised controlled trials: guidance on their use. PLoS Med 2015; 12: e1001855. 26196287 10.1371/journal.pmed.1001855 PMC4510878

[24] Trajman A, Durovni B, Saraceni V, Impact on patients’ treatment outcomes of Xpert MTB/RIF implementation for the diagnosis of tuberculosis: follow-up of a stepped-wedge randomized clinical trial. PLoS One 2015; 10: e0123252. 25915745 10.1371/journal.pone.0123252 PMC4411054

[25] Keeler E, Perkins MD, Small P, Reducing the global burden of tuberculosis: the contribution of improved diagnostics. Nature 2006; 444: 49–57. 10.1038/nature05446 17159894

[26] WHO. WHO monitoring of Xpert MTB/RIF roll-out http://www.who.int/tb/areas-of-work/laboratory/mtb-rif-rollout/en/ (accessed Dec 1, 2018).

[27] MacPherson P, Houben RM, Glynn JR, Corbett EL, Kranzer K. Pre-treatment loss to follow-up in tuberculosis patients in low- and lower-middle-income countries and high-burden countries: a systematic review and meta-analysis. Bull World Health Organ 2014; 92: 126–38. 24623906 10.2471/BLT.13.124800 PMC3949536

[28] Theron G, Peter J, Dowdy D, Langley I, Squire SB, Dheda K. Do high rates of empirical treatment undermine the potential effect of new diagnostic tests for tuberculosis in high-burden settings? Lancet Infect Dis 2014; 14: 527–32 24438820 10.1016/S1473-3099(13)70360-8

[29] Lawn SD, Nicol MP, Corbett EL. Effect of empirical treatment on outcomes of clinical trials of diagnostic assays for tuberculosis. Lancet Infect Dis 2015; 15: 17–18. 25541165 10.1016/S1473-3099(14)71049-7

[30] Calligaro GL, Zijenah LS, Peter JG, Effect of new tuberculosis diagnostic technologies on community-based intensified case finding: a multicentre randomised controlled trial. Lancet Infect Dis 2017; 17: 441–50. 28063795 10.1016/S1473-3099(16)30384-X

[31] Hermans S, Caldwell J, Kaplan R, Cobelens F, Wood R. The impact of the roll-out of rapid molecular diagnostic testing for tuberculosis on empirical treatment in Cape Town, South Africa. Bull World Health Organ 2017; 95: 554–63. 28804167 10.2471/BLT.16.185314 PMC5537747

[32] Braunholtz DA, Edwards SJ, Lilford RJ. Are randomized clinical trials good for us (in the short term)? Evidence for a “trial effect”. J Clin Epidemiol 2001; 54: 217–24. 11223318 10.1016/s0895-4356(00)00305-x

[33] Lessells RJ, Cooke GS, McGrath N, Nicol MP, Newell ML, Godfrey-Faussett P. Impact of point-of-care Xpert MTB/RIF on tuberculosis treatment initiation: a cluster randomised trial. Am J Respir Crit Care Med 2017; 196: 901–10. 28727491 10.1164/rccm.201702-0278OC PMC5649979

[34] Pocock SJ, Stone GW. The primary outcome fails—what next? N Engl J Med 2016; 375: 861–70. 27579636 10.1056/NEJMra1510064

[35] US Food and Drug Administration. Guidance for industry pulmonary tuberculosis: developing drugs for treatment 2013 https://www.fda.gov/downloads/Drugs/GuidanceComplianceRegulatoryInformation/Guidances/UCM373580.pdf (accessed Dec 22, 2017).

[36] Greenland S Nonsignificance plus high power does not imply support for the null over the alternative. Ann Epidemiol 2012; 22: 364–68. 22391267 10.1016/j.annepidem.2012.02.007

[37] Steingart KR, Schiller I, Horne DJ, Pai M, Boehme CC, Dendukuri N. Xpert(R) MTB/RIF assay for pulmonary tuberculosis and rifampicin resistance in adults. Cochrane Database Syst Rev 2014; 1: CD009593. 10.1002/14651858.CD009593.pub3 PMC4470349 24448973

[38] Andrews JR, Lawn SD, Rusu C, The cost-effectiveness of routine tuberculosis screening with Xpert MTB/RIF prior to initiation of antiretroviral therapy: a model-based analysis. AIDS 2012; 26: 987–95. 22333751 10.1097/QAD.0b013e3283522d47 PMC3517815

[39] Ngwira LG, Corbett EL, Khundi M, Screening for tuberculosis with Xpert MTB/RIF assay versus fluorescent microscopy among adults newly diagnosed with human immunodeficiency virus in rural Malawi: a cluster randomized trial (CHEPETSA). Clin Infect Dis 2018; published online July 27 DOI:10.1093/cid/ciy590. PMC6769397 30059995

[40] Debray TP, Moons KG, Abo-Zaid GM, Koffijberg H, Riley RD. Individual participant data meta-analysis for a binary outcome: one-stage or two-stage? PLoS One 2013; 8: e60650. 23585842 10.1371/journal.pone.0060650 PMC3621872

[41] Chakravorty S, Simmons AM, Rowneki M, The new Xpert MTB/RIF Ultra: improving detection of *Mycobacterium tuberculosis* and resistance to rifampin in an assay suitable for point-of-care testing. MBio 2017; 8: e00812–17. 28851844 10.1128/mBio.00812-17 PMC5574709

[42] Hayes RJ, Moulton LH. Cluster randomised trials Boca Raton, FL: CRC Press, 2009.

