# Supplementary material for: Effect of Xpert MTB/RIF on clinical outcomes in routine care settings: individual patient data meta-analysis
Source: Lancet Glob Health. Author manuscript; Available in PMC 2019 Feb 7. (PMC6366854; doi:10.1016/S2214-109X(18)30458-3)
Supplement: 1 [file NIHMS1519364-supplement-1.pdf]

THE LANCET
Global Health

Supplementary appendix

This appendix formed part of the original submission and has been peer reviewed. We post it as supplied by the authors.

Supplement to: Di Tanna GL, Khaki AR, Theron G, et al. Effect of Xpert MTB/RIF on clinical outcomes in routine care settings: individual patient data meta-analysis. *Lancet Glob Health* 2019; **7:** e191–99.

Appendix

Figure S1. Risk of bias assessment

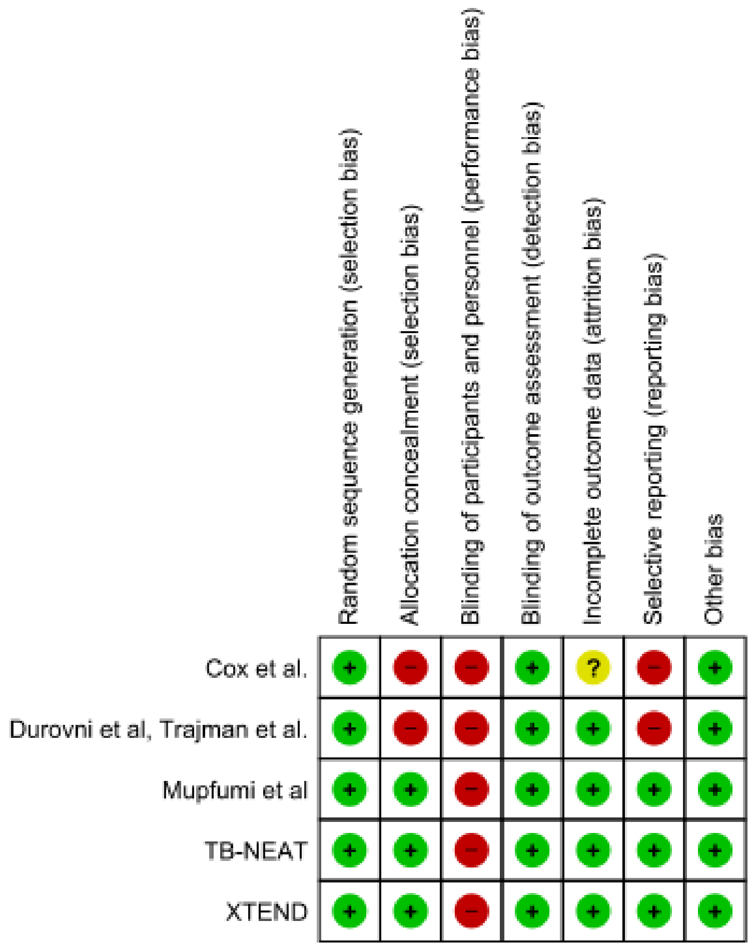

Table S1. Sensitivity analyses employing random effects and additional covariate adjustment

Adults investigated for TB	Studies[*]	Number of adults	OR/HR	95% CI		p (test of effect)	I^2	p (test of homogeneity)
**6-month mortality risk [†]**								
HIV-positive	[15, 17, 18]	4,066	0.83	0.64	1.08	0.24	14.8%	0.31
**Time to death**								
Overall, adjusted for age, gender, weight, TB symptoms	[15, 16, 18]	6,568	0.84	0.65	1.09	0.20	0.00%	0.41
HIV-positive, adjusted for age, gender, weight, TB symptoms	[15, 16, 18]	3,511	0.73	0.56	0.96	0.02	0.00%	0.64
**Time to TB diagnosis**								
Overall	[15, 16]	1,924	0.992	0.763	1.290	0.953	47.50%	0.167
HIV-positive	[15, 16]	1,317	0.977	0.790	1.208	0.827	25.20%	0.248
**3-month mortality risk**								
Overall, adjusted for age, gender, weight, TB symptoms [§]	[15, 16, 18]	6, 225	0.90	0.66	1.21	0.50	9.3%	0.33
Overall, adjusted for age, gender, weight, TB symptoms	[15, 16, 18]	6, 225	0.89	0.64	1.23	0.46	9.3%	0.33
**Time to TB treatment**								
Overall, adjusted for age, gender, weight, TB symptoms [§]	[15, 16, 18]	6,287	0.90	0.78	1.02	0.10	58.10%	0.09
Overall, adjusted for age, gender, weight, TB symptoms	[15, 16, 18]	6,287	0.89	0.71	1.12	0.32	58.10%	0.09
**Adults initiated on TB treatment**	**Studies[*]**	**Number of adults**	**OR/HR**	**95% CI**		**p (test of effect)**	**I^2**	**p (test of homogeneity)**
**Time to death**								

HIV-positive	[15, 16, 17, 18, 19]	1,445	0.85	0.52	1.39	0.46	18.00%	0.30

All estimates are adjusted for age and gender (unless additional covariates noted), and were combined using random effects regardless of heterogeneity (I^2) estimate (unless fixed effects are noted).

* References refer to the primary studies: [15] TB-NEAT; [16] Mupfumi et al.; [17] Cox et al.; [18] XTEND; [19] Durovni et al

† 6-month mortality risk was the primary outcome.

§ Analysed using fixed effects.

Definition of Abbreviations: OR, odds ratio; HR, hazard ratio; CI, confidence interval.

Table S2. Sensitivity analyses using the one-stage approach

Adults investigated for TB	Studies*	Number of adults	OR/HR	95% CI		p (test of effect)
**6-month mortality risk †**						
Overall	[15, 17, 18]	8,142	0.89	0.70	1.13	0.34
HIV negative	[15, 17, 18]	2,538	0.80	0.47	1.35	0.40
HIV positive	[15, 17, 18]	4,066	0.83	0.63	1.1	0.2
**Time to Death**						
Overall	[15, 16, 17, 18]	8,561	0.86	0.68	1.09	0.22
Overall, adjusted for age, gender, weight, TB symptoms	[15, 16, 18]	6,568	0.86	0.661	1.11	0.24
HIV negative	[15, 17, 18]	2,583	0.80	0.48	1.35	0.41
HIV positive	[15, 16, 17, 18]	4,486	0.81	0.63	1.05	0.11
HIV positive, adjusted for age, gender, weight, TB symptoms	[15, 16, 18]	3,511	0.76	0.57	1.01	0.06
No history of prior TB	[15, 16, 17, 18]	6,887	0.90	0.69	1.16	0.40
History of prior TB	[15, 16, 17, 18]	1,674	0.75	0.47	1.20	0.06
**3-month mortality risk**						
Overall	[15, 16, 17, 18]	8,566	0.92	0.71	1.20	0.53
Overall, adjusted for age, gender, weight, TB symptoms	[15, 16, 18]	6,572	0.90	0.68	1.19	0.46
HIV negative	[15, 17, 18]	2,583	0.76	0.43	1.35	0.35
HIV positive	[15, 16, 17, 18]	4,490	0.87	0.65	1.16	0.33
No history of prior TB	[15, 16, 17, 18]	6,891	1.02	0.77	1.37	0.88
History of prior TB	[15, 16, 17, 18]	1,675	0.59	0.34	1.02	0.06
**Time to TB diagnosis**						
Overall	[15, 16]	1,924	1.03	0.87	1.22	0.73

HIV positive	[15, 16]	1,317	0.98	0.80	1.20	0.87
**Time to TB treatment**						
Overall	[15, 16, 17, 18]	8,208	1.04	0.91	1.17	0.60
Overall, adjusted for age, gender, weight, TB symptoms	[15, 16, 18]	6,287	0.84	0.70	0.99	0.04
HIV negative	[15, 17, 18]	2,482	0.86	0.67	1.09	0.21
HIV positive	[15, 16, 17, 18]	4,251	1.07	0.92	1.25	0.39
No history of prior TB	[15, 16, 17, 18]	6,666	0.86	0.71	1.03	0.10
History of prior TB	[15, 16, 17, 18]	1,542	0.79	0.53	1.16	0.22

All pooled estimates are adjusted for age and gender, and are reported using a one-stage analytic approach.
* References refer to the primary studies: [15] TB-NEAT; [16] Mupfumi et al.; [17] Cox et al.; [18] XTEND; [19] Durovni et al
† 6-month mortality risk was the primary outcome.
Definition of Abbreviations: OR, odds ratio; HR, hazard ratio; CI, confidence interval.

Table S3. Summary of the trials contributing to the primary and secondary outcomes

	Theron et al. (TB-NEAT) [15]	Mupfumi et al. [16] *	Cox et al. [17]	Churchyard et al. (XTEND) [18]	Durovni et al., Trajman et al. [19,22]
**Adults investigated for TB**					
6-month mortality risk	X		X	X	
Time to death	X	X	X	X	
3-month mortality risk	X	X	X	X	
Time to TB diagnosis	X	X			
Time to starting TB treatment	X	X	X	X	
**Adults initiated on TB treatment**					
Time to death	X	X	X	X	X

[*] Contributed HIV-positive adults only.
